# Genomic Diversity of Methicillin-Resistant Staphylococcus aureus CC398 Isolates Collected from Diseased Swine in the German National Resistance Monitoring Program GE*RM*-Vet from 2007 to 2019

**DOI:** 10.1128/spectrum.00770-23

**Published:** 2023-05-08

**Authors:** Henrike Krüger-Haker, Xing Ji, Dennis Hanke, Stefan Fiedler, Andrea T. Feßler, Nansong Jiang, Heike Kaspar, Yang Wang, Congming Wu, Stefan Schwarz

**Affiliations:** a Institute of Microbiology and Epizootics, Centre for Infection Medicine, School of Veterinary Medicine, Freie Universität Berlin, Berlin, Germany; b Veterinary Centre for Resistance Research (TZR), School of Veterinary Medicine, Freie Universität Berlin, Berlin, Germany; c Jiangsu Key Laboratory for Food Quality and Safety, State Key Laboratory, Cultivation Base of Ministry of Science and Technology, Institute of Food Safety and Nutrition, Jiangsu Academy of Agricultural Sciences, Nanjing, China; d Federal Office of Consumer Protection and Food Safety (BVL), Berlin, Germany; e Key Laboratory of Animal Antimicrobial Resistance Surveillance, MARA, College of Veterinary Medicine, China Agricultural University, Beijing, China; Universität Greifswald

**Keywords:** LA-MRSA, evolution, antimicrobial resistance monitoring, whole-genome sequencing, cgMLST, antimicrobial susceptibility testing

## Abstract

Livestock-associated methicillin-resistant Staphylococcus aureus (LA-MRSA) clonal complex 398 (CC398) isolates (*n* = 178) collected in the national resistance monitoring program GE*RM*-Vet from diseased swine in Germany from 2007 to 2019 were investigated for their genomic diversity with a focus on virulence and antimicrobial resistance (AMR) traits. Whole-genome sequencing was followed by molecular typing and sequence analysis. A minimum spanning tree based on core-genome multilocus sequence typing was constructed, and antimicrobial susceptibility testing was performed. Most isolates were assigned to nine clusters. They displayed close phylogenetic relationships but a wide molecular variety, including 13 *spa* types and 19 known and four novel *dru* types. Several toxin-encoding genes, including *eta*, *seb*, *sek*, *sep*, and *seq*, were detected. The isolates harbored a wide range of AMR properties mirroring the proportions of the classes of antimicrobial agents applied in veterinary medicine in Germany. Multiple novel or rare AMR genes were identified, including the phenicol-lincosamide-oxazolidinone-pleuromutilin-streptogramin A resistance gene *cfr*, the lincosamide-pleuromutilin-streptogramin A resistance gene *vga*(C), and the novel macrolide-lincosamide-streptogramin B resistance gene *erm*(54). Many AMR genes were part of small transposons or plasmids. Clonal and geographical correlations of molecular characteristics and resistance and virulence genes were more frequently observed than temporal relations. In conclusion, this study provides insight into population dynamics of the main epidemic porcine LA-MRSA lineage in Germany over a 13-year-period. The observed comprehensive AMR and virulence properties, most likely resulting from the exchange of genetic material between bacteria, highlighted the importance of LA-MRSA surveillance to prevent further dissemination among swine husbandry facilities and entry into the human community.

**IMPORTANCE** The LA-MRSA-CC398 lineage is known for its low host specificity and frequent multiresistance to antimicrobial agents. Colonized swine and their related surroundings represent a considerable risk of LA-MRSA-CC398 colonization or infection for occupationally exposed people through which such isolates might be further disseminated within the human community. This study provides insight into the diversity of the porcine LA-MRSA-CC398 lineage in Germany. Clonal and geographical correlations of molecular characteristics and resistance and virulence traits were detected and may be associated with the spread of specific isolates through livestock trade, human occupational exposure, or dust emission. The demonstrated genetic variability underlines the lineage’s ability to horizontally acquire foreign genetic material. Thus, LA-MRSA-CC398 isolates have the potential to become even more dangerous for various host species, including humans, due to increased virulence and/or limited therapeutic options for infection control. Full-scale LA-MRSA monitoring at the farm, community, and hospital level is therefore essential.

## INTRODUCTION

Livestock-associated methicillin-resistant Staphylococcus aureus (LA-MRSA) has gained increasing attention as an important zoonotic pathogen since it was detected in Dutch swine in 2005 (1, 2). Clonal complex (CC) 398 is the major LA-MRSA lineage found in Europe, North and South America, and Australia (3–5). It has also been identified occasionally in Africa and Asia (6–8). CC398 comprises at least 43 sequence types (STs) (9), but among LA-MRSA, mainly ST398 isolates are described. Swine represent a major reservoir for LA-MRSA-CC398 (9). Such isolates are only occasionally reported as causes of diseases, such as pyoderma, pneumonia, or septicemia in swine, and mainly play a role as colonizers of the skin and the mucosal surfaces (3). However, LA-MRSA-CC398 has no particular host specificity. Thus, it can easily cross species barriers and colonize or infect other animals, such as cattle, sheep, goats, poultry, dogs, cats, horses, rabbits, rats, and mink, as well as humans (1, 10–16). In humans, LA-MRSA-CC398 isolates have mainly been reported from skin and soft tissue infections in the community and from nosocomial wound infections, pneumonia, and even septicemia when introduced into the hospital setting (1, 17). Persons in direct (occupational) contact with livestock are at particular risk of LA-MRSA-CC398 colonization or infection, and further human-to-human transmission cannot be excluded (1). It is important to note that the multiresistance to several classes of antimicrobial agents is typical for the LA-MRSA-CC398 lineage (9). Previous studies have shown that these isolates differ in their *spa* types, antimicrobial susceptibility profiles, and resistance and virulence gene patterns, highlighting the lineage’s diversity (3, 4, 18–20).

In this study, LA-MRSA-CC398 collected from diseased swine all over Germany during 13 years on the basis of one isolate per herd were comparatively investigated for their genomic diversity, with an emphasis on virulence and antimicrobial resistance (AMR) properties. Phylogenetic relationships, temporal and/or geographical accumulations of virulence, and AMR gene patterns, as well as the occurrence of mobile genetic elements (MGEs), such as small transposons or plasmids, were explored to understand the population dynamics within the main epidemic LA-MRSA lineage in Germany over time.

## RESULTS

### Molecular typing of 178 LA-MRSA-CC398 isolates revealed the presence of four STs, 13 *spa* types, 23 *dru* types, and two staphylococcal cassette chromosome *mec* element (SCC*mec*) types.

All STs detected were included in CC398, with the majority of isolates belonging to ST398 (*n* = 175; *arcC*_3, *aroE*_35, *glpF*_19, *gmk*_2, *pta*_20, *tpi*_26, *yqiL*_39). Three novel STs were submitted to the database, each differing in one allele from ST398 (ST6759, *n* = 1, *glpF*_45; ST7001, *n* = 1, *glpF*_884; ST7002, *n* = 1, *tpi*_779).

All *spa* and *dru* types identified are displayed in Table 1. The most common *spa* types were t011 (*n* = 120) and t034 (*n* = 38). Most *spa* types were closely related to each other, and t011 probably represented the original type from which the others have developed. In particular, single or multiple repeats were lost (t1451, t1456, t3423), and certain repeats were duplicated (t034, t2011, t2370) or exchanged (t1197). In other cases, most likely, an additional repeat was inserted (t2576), and repeats were deleted as well as duplicated (t1250) or lost and exchanged (t899, t2510, t6228). The most frequent *dru* types detected were dt11a (*n* = 117), dt6j (*n* = 13), and dt11af (*n* = 11). Four new *dru* types (dt8au, dt10ds, dt10dt, dt11dx) were submitted to the database. All *dru* types were closely related to one another, and dt11a most likely can be considered as the founder from which the others developed. Mostly, single or multiple repeats were exchanged (dt11ab, dt11af, dt11ag, dt11ap, dt11ax, dt11c, dt11ca, dt11cv, dt11dx, dt11v) or lost (dt3c, dt5e, dt6j, dt9v, dt10ao). However, repeats also seemed to have been deleted as well as exchanged (dt8ap, dt8au, dt10an, dt10dt, dt10q) or maybe even deleted, duplicated, and exchanged (dt10al, dt10ds).

**TABLE 1:** The *spa* and *dru* types observed among the 178 LA-MRSA-CC398 investigated in this study

*spa* type	Repeat order	No. of isolates	*dru* type	Repeat order	No. of isolates
t011	08-16-02-25-34-24-25	120	dt3c	5a-2d-3e	1
t034	08-16-02-25-02-25-34-24-25	38	dt5e	5a-2d-4a-0-3e	5
t899	07-16-23-02-34	1	dt6j	5a-2d-4a-0-2d-3e	13
t1197	08-16-02-25-46-24-25	2	dt8ap	5a-2d-4a-0-2d-5b-2a-2f	1
t1250	08-16-02-25-02-25	1	dt8au^*a*^	5a-2d-4a-0-2d-5b-2a-3e	1
t1451	08-16-02-25-34-25	3	dt9v	5a-2d-4a-0-2d-2g-3b-4e-3e	1
t1456	08-16-02-25	1	dt10al	5a-2d-2d-2d-5b-3a-2g-4b-4e-3e	6
t2011	08-16-16-02-25-34-24-25	1	dt10an	4k-4a-0-2d-5b-3a-2g-3b-4e-3e	3
t2370	08-16-16-02-25-02-25-34-24-25	1	dt10ao	5a-4a-0-2d-5b-3a-2g-3b-4e-3e	1
t2510	08-17-25	7	dt10ds^*a*^	5a-2d-2d-2d-5b-3a-2g-3c-4e-3e	1
t2576	08-12-16-02-25-34-24-25	1	dt10dt^*a*^	5a-2d-4a-0-2d-6f-3a-2g-3b-4e	1
t3423	08-16-02-25-34-24	1	dt10q	5a-2d-4a-0-2d-5b-3a-2g-2c-4e	1
t6228	35-25-34-24-25	1	dt11a	5a-2d-4a-0-2d-5b-3a-2g-3b-4e-3e	117
			dt11ab	5a-2d-4a-0-2d-5b-3a-2g-3b-4e-2f	3
			dt11af	5a-2d-4a-0-2d-5b-2a-2g-3b-4e-3e	11
			dt11ag	5a-3c-4a-0-2d-5b-2a-2g-3b-4e-3e	2
			dt11ap	5a-2d-4a-0-2d-5b-3a-3o-3b-4e-3e	1
			dt11ax	5a-2d-4a-0-2d-6f-3a-2g-3b-4e-3e	2
			dt11c	5a-2d-4a-0-2d-5b-3a-2g-4b-4e-3e	3
			dt11ca	5a-3c-4a-0-2d-5b-3a-2g-3b-4e-3e	1
			dt11cv	5a-2d-4a-1b-2d-6f-3a-2g-3b-4e-3e	1
			dt11dx^*a*^	5a-2d-4a-2d-2d-5b-2a-2g-3b-4e-3e	1
			dt11v	5a-2d-4a-0-3c-5b-3a-2g-3b-4e-3e	1

a Novel *dru* types identified within this study and submitted to the *dru* typing database.

In 174 isolates, SCC*mec* type Vc(5C2&5) cassettes were detected. One isolate was not subtypeable [SCC*mec* type V(5C2&5)], and another harbored an SCC*mec* type IVa(2B) element. Two isolates carried a truncated SCC*mec* cassette.

### The LA-MRSA-CC398 carried a uniform set of virulence genes typically associated with S. aureus.

All individual virulence gene profiles are given in Data set S1 in the supplemental material. Most importantly, the Panton-Valentine leucocidin (PVL) genes *lukF-PV* and *lukS-PV* and the toxic shock syndrome (TSS) toxin 1 gene *tst* were not identified. Moreover, none of the isolates carried the genes *sak*, *chp*, and *scn*, which are indicative of β-hemolysin-converting phages. All isolates also lacked the exfoliative toxin genes *etb* and *etd*. In contrast, they harbored *eta* and the exotoxin genes *set1*, *set4*, and *set5* as well as several hemolysin genes. The staphylococcal enterotoxin type P gene *sep* was also detected in all LA-MRSA isolates. However, the enterotoxin type B gene *seb* was additionally detected in only four isolates and another four isolates were additionally positive for enterotoxin type K and Q genes *sek* and *seq*. Furthermore, the isolates were negative for the protease genes *splA*, *splB*, and *splE*, but *sspA* (*n* = 176) and *sspB* (*n* = 178) were detected almost uniformly among all isolates. The protease-associated genes *hysA*^VSaβ^, *paiB*, and *cfim* were also identified in all LA-MRSA. Minor differences were detected regarding genes coding for microbial surface components recognizing adhesive matrix molecules (MSCRAMMs) such as *clfA* (*n* = 177), *clfB* (*n* = 171), *cna* (*n* = 154), *ebh* (*n* = 173), *efb* (*n* = 178), *fnbA* (*n* = 171), *fnbB* (*n* = 176), *sdrC* (*n* = 169), *sdrD* (*n* = 174), and *sdrE* (*n* = 176). All isolates carried a chromosomal *icaRADBC* operon for biofilm formation. Moreover, 34 isolates harbored an additional *ica*-like gene cluster, most likely on a plasmid. Seven types were identified with the majority of LA-MRSA (*n* = 20) harboring an *ica*-like operon that was described before on plasmid pAFS11 from a bovine MRSA-ST398 isolate (21). Plasmid pHKS3860 was identified in isolate 4 and harbored a singular type of *ica*-like gene cluster (22). Finally, the von Willebrand factor-binding protein gene *vwb* was identified (*n* = 177), and all isolates shared capsule type 5.

### Numerous AMR genes were detected, many of which were carried by small transposons or plasmids.

The AMR genes that were found are listed in Table 2 (23), and individual AMR gene profiles are shown in Data set S1. The LA-MRSA carried 3 to 15 different AMR genes, and 158 isolates showed multiresistance (24). All isolates harbored at least one β-lactam resistance gene (*mecA*) and one tetracycline resistance gene. A single isolate had two *blaZ* copies. In several isolates, combinations of genes conferring resistance to members of the same class (Table 2) or to different classes of antimicrobial agents were found. Interestingly, seven isolates harbored the genes *lnu*(B) plus *lsa*(E) plus *aadD* plus *aadE* plus *spw* in combination. The combination of *lnu*(B) plus *lsa*(E) also occurred in another two isolates. In all *apmA*-carrying isolates, *aadD* was also detected. Another isolate was positive for both *fexA* and *cfr*. Besides AMR genes, fluoroquinolone resistance-mediating point mutations in *gyrA* and *grlA* were detected, which resulted in the amino acid exchanges S84L/S80F (*n* = 8) and S84L/S80Y (*n* = 13), respectively.

**TABLE 2:** Antimicrobial resistance genes harbored by the 178 LA-MRSA-CC398 included in the study

Resistance to:	Gene	No. of isolates	Gene combinations	No. of isolates
β-Lactams	*blaZ*	172^*a*^	*blaZ* + *mecA*	172
	*mecA*	178		
Tetracyclines	*tet*(K)	172	*tet*(K) + *tet*(M)	131
	*tet*(L)	43	*tet*(L) + *tet*(M)	3
	*tet*(M)	177	*tet*(K) + *tet*(L) + *tet*(M)	40
	*tet*(38)	178^*b*^		
Macrolides, lincosamides, streptogramin B	*erm*(A)	23	*erm*(A) + *erm*(B)	5
	*erm*(B)	39	*erm*(A) + *erm*(C)	4
	*erm*(C)	48	*erm*(A) + *erm*(54)	1
	*erm*(T)	10	*erm*(B) + *erm*(C)	2
	*erm*(54)	1	*erm*(C) + *erm*(T)	3
			*erm*(A) + *erm*(B) + *erm*(C)	1
Lincosamides	*lnu*(A)	3		
	*lnu*(B)	9		
Lincosamides, pleuromutilins, streptogramin A	*vga*(A)_V_	25		
	*vga*(A)_LC_	2		
	*vga*(C)	1		
	*vga*(E)	21		
	*lsa*(E)	9		
Aminoglycosides	*aacA*-*aphD* (gentamicin, kanamycin, tobramycin, amikacin)	15	*aacA-aphD* + *str*	1
			*aacA*-*aphD* + *aadD* + *str*	9
	*aadD* (kanamycin, neomycin, tobramycin)	42	*aadD* + *aadE*	5
			*aadD* + *str*	20
	*aadE* (streptomycin)	7	*aadD* + *aadE* + *str*	2
	*str* (streptomycin)	82		
Aminocyclitols	*spc* (spectinomycin)	24	*spc + apmA*	1
	*spc*_V_ (spectinomycin)	2	*spc + spw + apmA*	1
	*spd* (spectinomycin)	2		
	*spw* (spectinomycin)	7		
	*apmA* (apramycin, decreased gentamicin susceptibility)	20		
Trimethoprim	*dfrG*	47		
	*dfrK*	47		
Phenicols	*fexA*	16		
Phenicols, lincosamides, oxazolidinones, pleuromutilins, streptogramin A	*cfr*	1		

a One isolate harbored two *blaZ* copies.

b The *tet*(38) gene can be found in nearly every S. aureus genome, including phenotypically susceptible isolates, and may confer resistance to tetracycline only when overexpressed (23).

**FIG 1:**
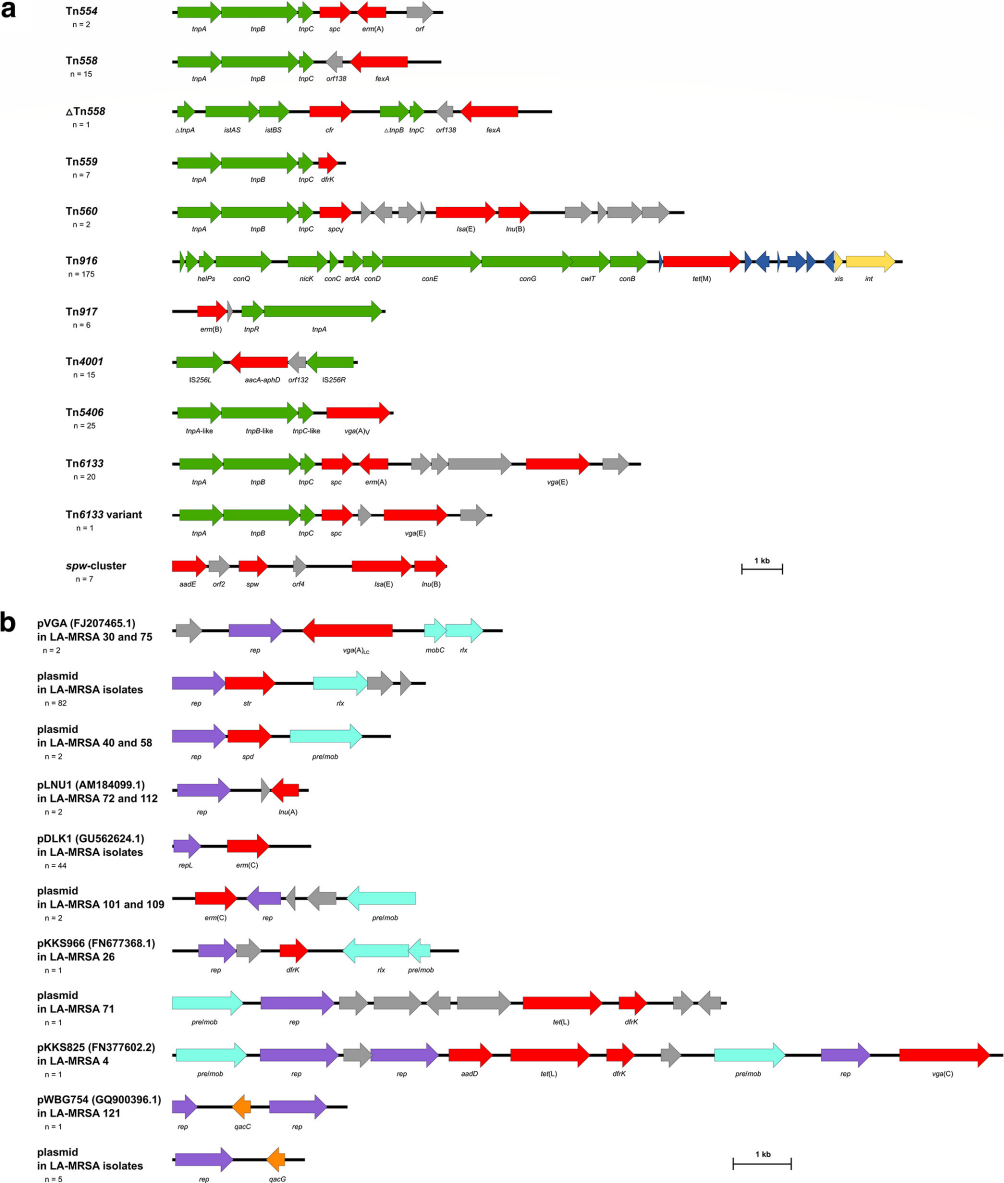
(a and b) Schematic comparison of (a) small transposons and the *spw*-cluster (25–37) and (b) small plasmids carried by the porcine LA-MRSA-CC398 from Germany. The number of isolates that harbored each mobile genetic element (MGE) is given below its designation. In cases where direct matches were found with plasmids from the database, the accession number is given in parentheses after the plasmid designation. The open reading frames are shown as arrows with the arrowhead indicating the direction of transcription. The transposase/conjugative transfer genes, recombination genes, plasmid recombination/mobilization genes, and replication genes are shown in green, yellow, turquoise, and purple, respectively. The antimicrobial resistance (AMR) genes, biocide resistance genes, transcriptional regulation genes, and genes encoding other functions are displayed in red, orange, dark blue, and gray, respectively. Truncated genes are indicated by a triangle, and size scales are given in the bottom-right corners.

Figure 1a shows that several AMR genes were part of small transposons or the multiresistance *spw* cluster, which is most likely of enterococcal origin (25–37). Overall, all *lnu*(B), *vga*(A)_V_, *lsa*(E), *aadE*, *spc*_V_, *spw*, *fexA*, and *cfr* genes identified here were found to be part of such an MGE. Only a fragmented Tn*916* was detected in two *tet*(M)-carrying isolates. One *vga*(E)-carrying isolate harbored a variant of Tn*6133*, which lacked *erm*(A) and three further open reading frames. Tn*4001* is only 4.5 kb in size and comprises an *aacA-aphD* gene flanked by two IS*256* insertion sequence elements. Thus, the complete Tn*4001* could not be identified based on Illumina short-read sequences. Nevertheless, *aacA-aphD* was intact in all 15 isolates carrying this gene. However, the presence of the complete Tn*4001* in one isolate with a closed genome also suggested its presence in the remaining 14 isolates. Besides a complete *fexA*-carrying transposon, Tn*558*, detected in 15 isolates, a truncated ΔTn*558* in which the transposase genes Δ*tnpA* and Δ*tnpB* were partly deleted and replaced by the genes *istAS*, *istBS*, and *cfr*, was identified in a single isolate.

Several AMR genes were detected on small plasmids of 2,360 to 5,713 bp in size, including all *vga*(A)_LC_, *str*, and *spd* genes as well as almost all *erm*(C) and *lnu*(A) genes of the LA-MRSA collection. In one and two isolate(s), the genetic environment of *lnu*(A) and *erm*(C), respectively, was fragmented. The *dfrK* gene was also identified on a small plasmid of 5,594 bp in one isolate. In addition, *dfrK* was found on a 9,585-bp plasmid with *tet*(L) in one isolate and on a 14,362-bp plasmid in combination with *aadD*, *vga*(C), and *tet*(L) in another isolate. A 36,929-bp plasmid harboring *erm*(54) next to an *ica* locus, the cadmium resistance genes *cadD* and *cadX*, the mercury resistance genes *merR* and *merA*, and the copper resistance genes *copB* and *mco* was described in one isolate (22). Moreover, six isolates harbored a small plasmid of 2,216 to 2,295 bp that carried a biocide resistance gene, either *qacC* (*n* = 1) or *qacG* (*n* = 5), both mediating elevated MICs to quaternary ammonium compounds such as benzalkonium chloride. In accordance with a previous study that described *qacC*-harboring S. aureus isolates from primates, which showed a slightly elevated MIC of 0.0004% for benzalkonium chloride (38), these isolates exhibited MIC values of 0.0005 to 0.001% for this biocide. Representative plasmid maps are shown in Fig. 1b.

### The genotypic AMR profiles correlated almost completely with the phenotypic ones.

Antimicrobial susceptibility testing results are displayed in Table 3. A total of 67 phenotypic AMR profiles were observed (Data set S1). For one *erm*(A)-positive isolate, inducible macrolide/lincosamide resistance was detected. MIC values of linezolid and quinupristin-dalfopristin were not available for all LA-MRSA, but in one isolate they confirmed expression of the *cfr* gene. Despite the presence of an AMR gene in the genome, the associated phenotype was not detected in one *fexA*-carrying, three *lnu*(A)-carrying, and eight *vga*(A)_V_-carrying isolates. Changes in neither the nucleotide sequences of the respective resistance genes nor the associated promoter regions were found. In addition, four of 13 isolates harboring the S84L/S80Y exchanges in GyrA and GrlA showed MIC values lower than those considered resistant (Table 3). No resistance-mediating genes or point mutations were identified in two isolates exhibiting resistance to erythromycin and gentamicin, respectively.

**TABLE 3:** Distribution of MIC values for the 178 LA-MRSA-CC398 isolates identified in this study

Antimicrobial agent(s)	No. of isolates with MIC (mg/L) of:																	Isolates that are:					
																		Susceptible		Intermediate		Resistant^*a*^	
	0.008	0.015	0.03	0.06	0.12	0.25	0.5	1	2	4	8	16	32	64	128	256	512	*n*	%	*n*	%	*n*	%
Oxacillin	X	-	-	-	-	-	-	-	-	13	71	*94*	X	X	X	X	X	-	**-**			178	**100**
Penicillin^*b*^	X	-	-	-	-	-	-	1	-	4	21	83	43	*26*	X	X	X	-	**-**			178	**100**
Ampicillin^*b*^	X	X	-	-	-	-	-	1	-	13	48	75	24	14	*3*	X	X	-	**-**			178	**100**
Amoxicillin/clavulanic acid^*b*^^,^^*c*^	X	X	-	-	-	-	-	1	21	100	46	9	1	-	X	X	X	-	**-**			178	**100**
Ceftiofur^*b*^	X	X	-	-	-	-	-	-	2	86	62	21	4	2	*1*	X	X	-	**-**			178	**100**
Cefquinome^*b*^	X	-	-	-	-	-	-	7	90	59	21	1	-	X	X	X	X	-	**-**			178	**100**
Cefotaxime^*b*^	X	-	-	-	-	-	-	-	-	2	92	62	19	*3*	X	X	X	-	**-**			178	**100**
Cefoperazone^*b*^	X	X	X	-	-	-	-	-	-	-	91	66	17	*4*	X	X	X	-	-			178	**100**
Erythromycin	X	-	-	-	-	23	47	1	1	1	-	1	-	*104*	X	X	X	70	**39**	3	**2**	105	**59**
Tylosin tartrate	X	X	X	-	-	-	-	58	26	1	1	1	-	-	-	*91*	X						
Tilmicosin	X	X	X	-	-	-	1	70	5	4	3	1	-	-	-	94	X						
Clindamycin	X	X	-	3	47	16	4	-	-	-	11	4	1	-	*92*	X	X	70	**39**	-	**-**	108	**61**
Pirlimycin	X	X	-	-	-	12	44	12	1	-	1	8	8	-	*92*	X	X						
Enrofloxacin		-	-	3	84	48	6	14	6	14	3	-	X	X	X	X	X	161	**90**	-	**-**	17	**10**
Gentamicin	X	X	X	X	-	51	75	14	1	11	10	-	5	8	3	-	X	152	**85**	10	**6**	16	**9**
Tetracycline	X	X	X	X	-	-	-	-	-	-	-	-	1	10	167	-	X	-	**-**	-	**-**	178	**100**
Sulfamethoxazole/trimethoprim^*c*^	X	-	2	65	17	16	39	22	9	8	-	-	-	X	X	X	X	170	**96**			8	**4**
Vancomycin	X	-	-	-	-	-	94	82	2	-	-	-	-	X	X	X	X	178	**100**	-	**-**	-	**-**

a Isolates were classified as susceptible, intermediate, or resistant if breakpoints were available in the CLSI document VET01S or M100 (97, 98). Despite the lack of breakpoints, isolates that showed high MIC values of enrofloxacin (≥4 mg/L) were considered resistant (3).

b Since oxacillin-resistant staphylococci are resistant to all currently available β-lactams, except for ceftaroline, resistance to these agents can be deduced from the oxacillin MIC values (97, 98).

c MIC values of amoxicillin/clavulanic acid (2:1) and sulfamethoxazole/trimethoprim (19:1) are given as amoxicillin and trimethoprim MIC values, respectively. Concentrations not included in the test panel are marked with an X or italic text. Isolates displayed in the lowest not-tested concentration showed growth in the highest test concentration, and the MIC is equal to or greater than the concentration following the highest test concentration. Black vertical lines indicate, if available, the CLSI breakpoints used to classify the isolates as susceptible, intermediate (if available), or resistant.

### The LA-MRSA-CC398 displayed close phylogenetic relationships.

**FIG 2:**
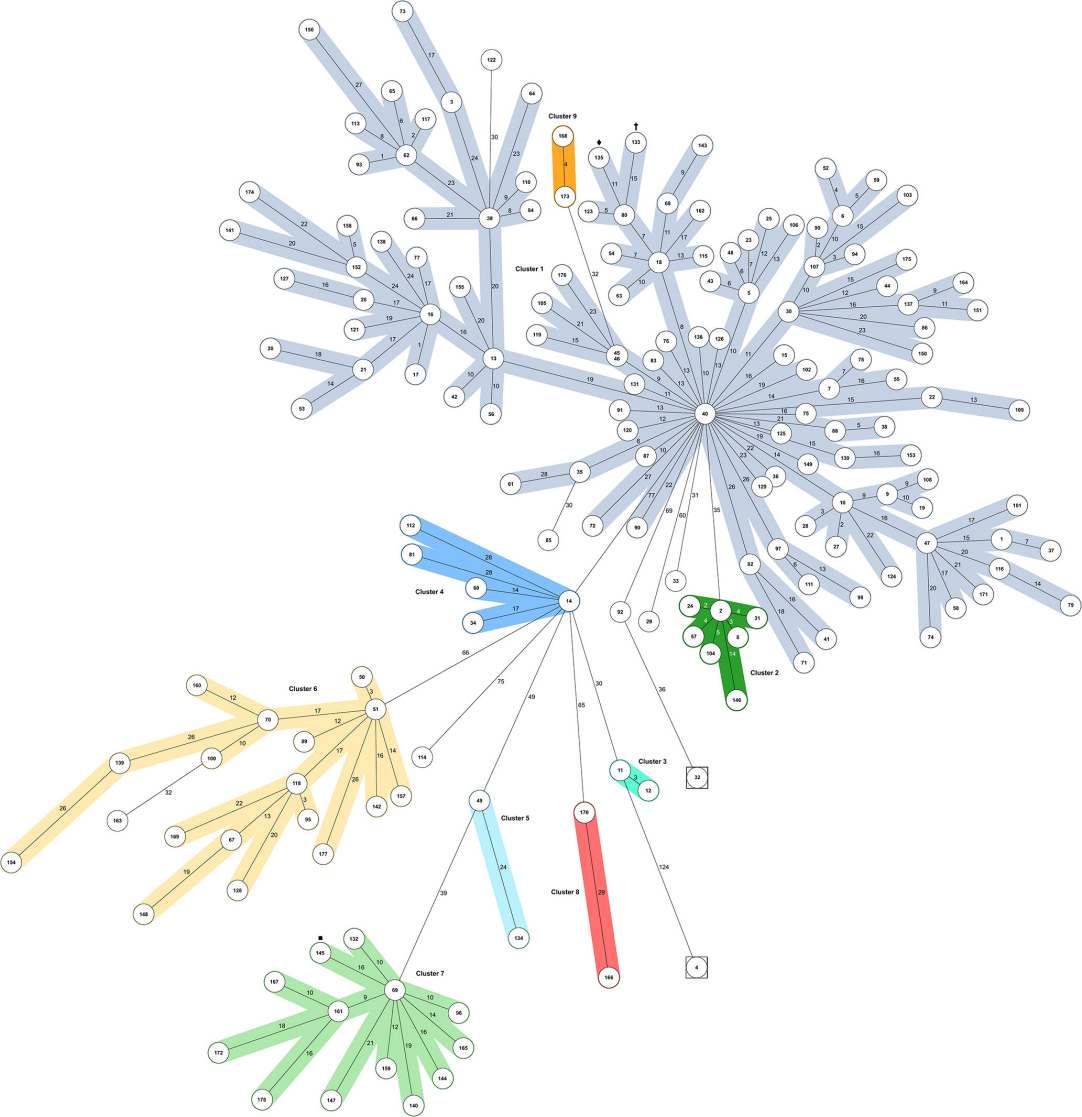
Minimum spanning tree displaying the phylogenetic relationship of the 178 LA-MRSA-CC398 isolates from Germany based on core-genome multilocus sequence typing (cgMLST) analysis with SeqSphere+, including 1,569 alleles. The circles represent different allelic profiles, and the count of varying target genes between them is shown next to the connecting lines. The nine clusters are illustrated in different colors: cluster 1 in blue-gray, cluster 2 in dark green, cluster 3 in turquoise, cluster 4 in dark blue, cluster 5 in light blue, cluster 6 in yellow, cluster 7 in light green, cluster 8 in red, and cluster 9 in orange. The two isolates carrying a truncated SCC*mec* cassette are framed by a box. The three isolates not belonging to sequence type (ST) 398 are marked by a rhombus (ST6759), square (ST7001), or cross (ST7002).

**FIG 3:**
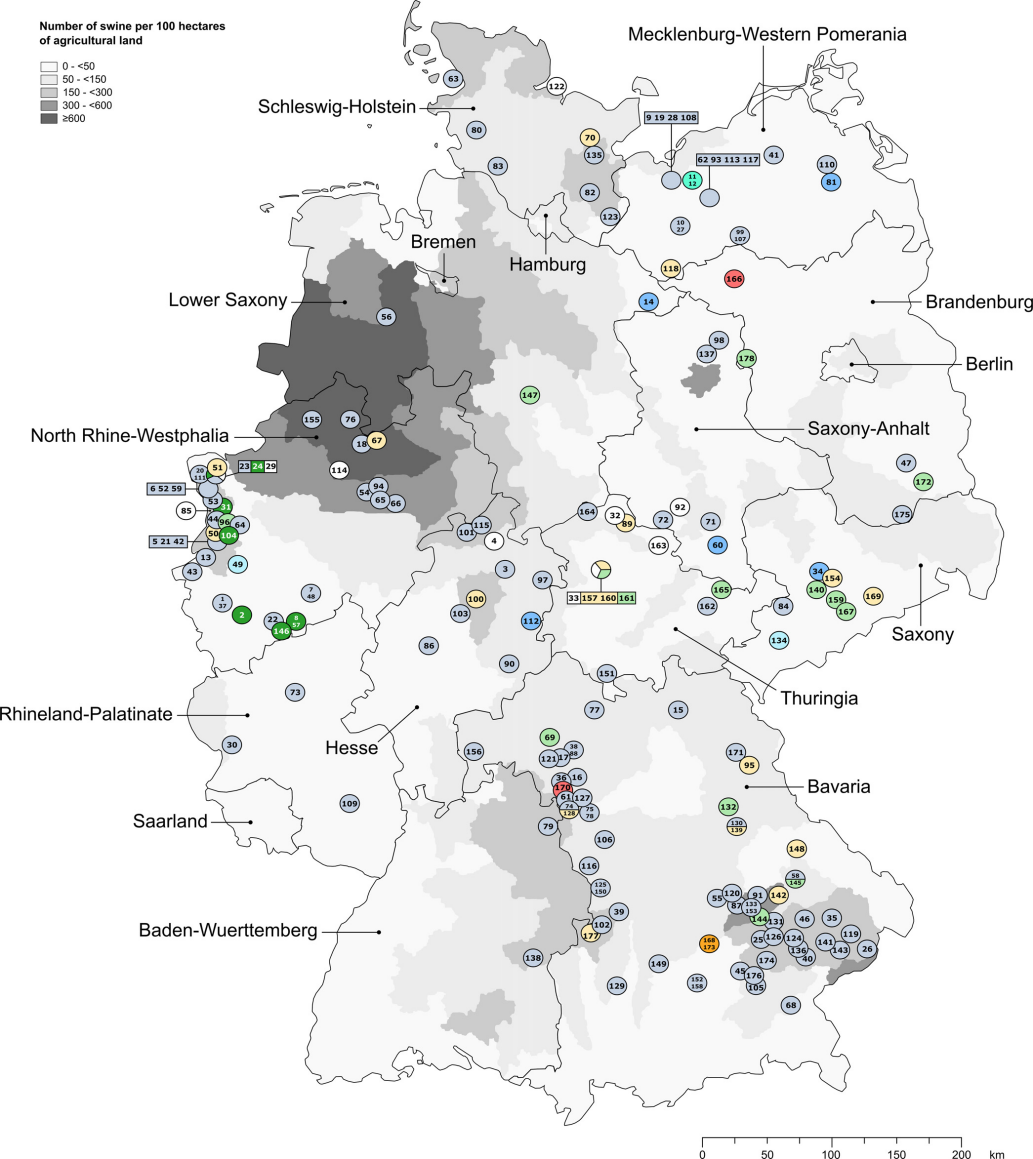
Swine density in Germany for the year 2020 and approximate locations of the farms from which the LA-MRSA-CC398 isolates originated based on zip code regions. The swine density is given as the number of swine per 100 ha of agricultural land according to the German federal and state statistical offices (https://agraratlas.statistikportal.de/#). A color legend is given in the top-left corner, and a distance scale is displayed in the bottom-right corner. The isolate IDs are given within the circles or rectangles, which contain more than one number in cases where multiple isolates were found within the same zip code area. The various colors of the circles and rectangles refer to the differentiation of isolate clusters shown by the minimum spanning tree in Fig. 2: cluster 1 in blue-gray, cluster 2 in dark green, cluster 3 in turquoise, cluster 4 in dark blue, cluster 5 in light blue, cluster 6 in yellow, cluster 7 in light green, cluster 8 in red, and cluster 9 in orange.

The minimum spanning tree based on core-genome multilocus sequence typing (cgMLST) revealed 177 allelic profiles comprising 9 clusters and 9 singletons (Fig. 2). Isolates 45 and 46 shared a profile. The profiles varied in 0 to 156 target genes of 1,569 alleles included (Data set S2, Fig. 2), and isolates were assigned to clusters with a maximum threshold of 29 allelic differences. Cluster 1 represented the main cluster comprising 119 isolates and started to emerge in 2007. Clusters 2 and 3 also developed starting in 2007 and finally consisted of seven and two isolates, respectively. Clusters 4, 5, and 6, including 5, 2, and 17 isolates, respectively, began to arise in 2008. Cluster 7 comprised 13 isolates and was first identified in 2009. Most recently, clusters 8 and 9 started to emerge in 2018, and both included two isolates. The relation between phylogenetic clustering and spatial distribution of the LA-MRSA-CC398 is demonstrated in Fig. 3 and Table 4.

**TABLE 4:** Isolates identified by federal state and year, including the affiliation to a cluster

Federal state^*a*^ (no. of isolates/state in total)	Yr^*b*^	Isolate ID(s)	Isolates within the same zip code regions	Cluster(s)
Baden Wuerttemberg (2)	2009	79		1
	2016	138		1
Bavaria (63)	2008	15, 16, 17, 25, 26, 35, 36, 38, 39, 40, 45, 46, 55, 58		1
	2009	61, 68, 69, 74, 75, 77, 78	75 and 78	1, 7
	2010	87, 88, 91, 95, 102, 105, 106, 116		1, 6
	2012	119, 120, 121		1
	2013	124, 125, 126, 127, 128, 129, 130, 131, 132, 133		1, 6, 7
	2015	136		1
	2016	139, 141, 142, 143, 144, 145, 148, 149, 150, 152, 153, 156, 158	152 and 158	1, 6, 7
	2018	168, 170, 171, 173, 174	168 and 173	1, 8, 9
	2019	176, 177		1, 6
Brandenburg (5)	2008	47		1
	2018	166, 172		7, 8
	2019	175, 178		1, 7
Hesse (10)	2007	3, 4		1, singleton
	2010	86, 90, 97, 100, 101, 103, 112, 115		1, 4, 6
Lower Saxony (3)	2008	14, 56		1, 4
	2016	147		7
Mecklenburg-Western Pomerania (18)	2007	9, 10, 11, 12, 19	9 and 19; 11 and 12	1, 3
	2008	27, 28, 41		1
	2009	62, 81		1, 4
	2010	93, 99, 107, 108, 110, 113	93 and 113; 99 and 107	1
	2011	117, 118		1, 6
North Rhine-Westphalia (41)	2007	1, 2, 5, 6, 7, 8		1, 2
	2008	13, 18, 20, 21, 22, 23, 24, 29, 31, 37, 42, 43, 44, 48, 49, 50, 51, 52, 53, 54, 57	21 and 42; 23, 24, and 29	1, 2, 5, 6, singleton
	2009	59, 64, 65, 66, 67, 76		1, 6
	2010	85, 94, 96, 104, 111, 114		1, 2, 7, singletons
	2016	146, 155		1, 2
Rhineland-Palatinate (3)	2008	30		1
	2009	73		1
	2010	109		1
Saxony (8)	2008	34		4
	2010	84		1
	2013	134		5
	2016	140, 154, 159		6, 7
	2018	167, 169		6, 7
Saxony-Anhalt (6)	2009	60, 71, 72		1, 4
	2010	92, 98		1, singleton
	2015	137		1
Schleswig-Holstein (8)	2009	63, 70, 80		1, 6
	2010	82, 83		1
	2012	122		Singleton
	2013	123		1
	2014	135		1
Thuringia (11)	2008	32, 33		Singletons
	2010	89		6
	2016	151, 157		1, 6
	2017	160, 161, 162	160 and 161	1, 6, 7
	2018	163, 164, 165		1, 7, singleton

a No isolates were detected in the federal states Berlin, Bremen, Hamburg, and Saarland.

b Numbers of isolates detected per year in total: 2007, *n* = 13; 2008, *n* = 45; 2009, *n* = 23; 2010, *n* = 35; 2011, *n* = 2; 2012, *n* = 4; 2013, *n* = 12; 2014, *n* = 1; 2015, *n* = 2; 2016, *n* = 22; 2017, *n* = 3; 2018, *n* = 12; 2019, *n* = 4.

### Isolates within a cluster shared specific characteristics, and three clusters showed geographical correlations.

The 119 isolates associated with the largest cluster, cluster 1, were found all over Germany during the whole study period. Remarkably, 114 of them displayed the dominant *spa* type, t011. Three isolates had *spa* type t1451, and one isolate each *spa* type t2011 or t2576, all present exclusively in cluster 1. The second most common *spa* type, t034 (*n* = 38), was not found within cluster 1, but was found in clusters 3 to 8 and among the singletons. Several AMR genes were exclusively observed within cluster 1, including *erm*(T), *vga*(A)_LC_ on a plasmid, *aacA-aphD*, *apmA*, *spd* on a plasmid, and *cfr* as part of ΔTn*558* (see Table 2). Moreover, most of the *tet*(L)-, *erm*(B)-, *erm*(C)-, and *dfrK*-carrying isolates (36/43, 27/39, 31/48 [on plasmid], and 38/47, respectively) were found within cluster 1, while only four out of 23 *erm*(A)-harboring and five out of 47 *dfrG*-harboring isolates were detected here. Almost all *fexA*-carrying isolates (13/16) were also associated with this cluster, with 12 isolates harboring Tn*558* and one harboring ΔTn*558*. Moreover, the *qacC*-positive isolate and three of five *qacG*-carrying LA-MRSA were part of cluster 1.

The seven cluster 2 isolates originated exclusively from North-Rhine Westphalia, near the Dutch border, with two isolates from the same zip code area. The *spa* (t2510) and *dru* (dt10al, dt10ds) types detected were unique within the LA-MRSA collection. The *vga*(A)_V_ gene as part of Tn*5406* was detected in all seven isolates. They showed a multiresistance profile harboring five to 10 AMR genes. Six of them carried *erm*(B), *aadD*, *tet*(K), and *tet*(L) as well as *tet*(M) on Tn*916*, which was, however, in one isolate only available fragmented. Five isolates harbored a *dfrK* gene. Moreover, three of four isolates positive for the enterotoxin genes *sek* and *seq* were associated with cluster 2.

Two isolates assigned to cluster 3 were collected in 2007 within the same zip code region in Mecklenburg-Western Pomerania. They were multiresistant and had an identical AMR gene profile, including *blaZ*, *mecA*, *tet*(K), *tet*(M) as part of Tn*916*, *erm*(C) on a plasmid, and *dfrG*.

All five cluster 4 isolates were collected relatively far apart from each other from 2008 to 2010 in central and northern Germany. They harbored AMR genes conferring resistance to three to five classes of antimicrobial agents and, thus, were classified as multiresistant. Three isolates harbored *vga*(A)_V_ as part of Tn*5406*, and all isolates were positive for *dfrG*.

Although the two cluster 5 isolates originated from very different locations (North-Rhine Westphalia/Saxony) and years (2008/2013), they carried the same AMR genes (*blaZ*, *mecA*, *tet*(K), Tn*916* with *tet*(M), *str* on a plasmid, *dfrG*).

A wide distribution over Germany was also seen for the 17 cluster 6 isolates collected during almost the complete study period. Interestingly, all five isolates with *dru* type dt5e and 12 of 13 isolates with *dru* type dt6j were observed within this cluster. With eight to 11 AMR genes, the isolates harbored a particularly large number of resistance genes. The multiresistance profiles were almost identical [*blaZ*, *mecA*, *tet*(K), Tn*916* with *tet*(M)—fragmented in one isolate, *erm*(A), *erm*(B), *erm*(C), *vga*(E), *str* on a plasmid, *spc*, *dfrG*; individual losses of *erm*(B) and/or *erm*(C) and/or *str*]. In these isolates, 17 of 23 *erm*(A) genes were detected as part of Tn*6133* with *vga*(E) and *spc*, alone or in combination with either *erm*(B) found on Tn*917* (6/6 isolates) or *erm*(C) on a plasmid. Considering *vga*(E) and *spc*, 17 out of 21 and 24 positive isolates, respectively, were identified within this cluster.

Cluster 7 comprised 13 isolates identified at greater distances to each other mainly in central and southern Germany. Interestingly, 10 of 11 *dru* type dt11af isolates were part of this cluster. Seven isolates carried *erm*(C) on a plasmid, and three isolates carried *fexA* as part of Tn*558*. The same six isolates harbored, next to *aadD*, the *spw*-cluster that also comprised *lnu*(B), *lsa*(E), and *aadE*. This combination was otherwise only identified in a single cluster 1 isolate. Moreover, all cluster 7 isolates were positive for *dfrG* and the same kind of variations in GyrA and GrlA (S84L/S80Y), which were not detected in other isolates.

The two cluster 8 isolates from 2018 differed not only in their origin (Brandenburg/Bavaria), but also in their AMR gene profiles. However, both isolates exhibited a multiresistance profile, including *dfrG* and Tn*560* comprising *lnu*(B), *lsa*(E), and *spc*_V_. The *spc*_V_ gene was not found in other isolates.

The two cluster 9 isolates from 2018 originated from the same zip code region in Bavaria. They carried only the AMR genes *blaZ*, *mecA*, *tet*(K), and, as part of Tn*916*, *tet*(M) and were resistant only to β-lactams and tetracyclines.

Eight of the remaining nine singletons were isolated in central Germany, while one isolate came from northern Schleswig-Holstein. The only isolate of the collection harboring *vga*(C) [on a plasmid with *aadD*, *dfrK*, and *tet*(L)] and *erm*(54) (on another plasmid with heavy metal resistance genes) was found among these singletons. Considering trimethoprim resistance, *dfrG* and *dfrK* were detected here. Three of eight isolates positive for one or two enterotoxin gene(s) in addition to *sep* also belonged to the singletons.

### Locations of the farms from which the LA-MRSA-CC398 isolates originated largely reflected the pork production density in Germany, and further geographical correlations were revealed.

Most of the 178 isolates were collected in southern Germany (Bavaria, *n* = 63) and western Germany near the Dutch border (North-Rhine Westphalia, *n* = 41) (Fig. 3, Table 4).

The *spa* type t1197 was detected in two isolates found relatively close to each other (32 and 92, Fig. 3). Moreover, two of three *spa* type t1451 isolates were collected close to each other in Bavaria (16 and 17, Fig. 3). All seven *spa* type t2510 isolates were identified exclusively in North-Rhine Westphalia grouped within cluster 2 as mentioned above. Six of these isolates displayed *dru* type dt10al, which was not observed more often. Two of three *dru* type dt11ab isolates originated from relatively close locations on the border between Baden-Württemberg and Bavaria (79 and 116, Fig. 3).

One isolate carrying the enterotoxin gene *seb* and all four isolates positive for the enterotoxin genes *sek* and *seq* were detected in proximity to each other in North-Rhine Westphalia (155/29, 31, 57, and 104, Fig. 3).

The most frequently detected AMR genes within our collection were spread throughout Germany. However, isolates harboring specific genes still aggregated partly in particular regions. Furthermore, some genes were found in nearby isolates but also in single LA-MRSA isolates from more distant areas. AMR genes that occurred less frequently were often found in isolates of more distant geographical origin. Interestingly, *aacA*-*aphD* was mainly identified in isolates from Mecklenburg-Western Pomerania (9, 10, 19, 27, 28, 108) and Bavaria (58, 74, 61, 116, 124, 171) (Fig. 3). Four and two isolates, respectively, from Mecklenburg-Western Pomerania even originated from the same zip code region. Out of 20 *apmA*- and *aadD*-positive isolates, five (63, 80, 83, 123, 135; Fig. 3) originated from Schleswig-Holstein, and six of these 20 isolates (44, 52, 54, 59, 76, 94; Fig. 3) originated from North-Rhine Westphalia. Only two isolates (40 and 58) harbored *spd* on a plasmid, but both were identified relatively nearby in Bavaria (Fig. 3). Out of 16 isolates carrying *fexA* on Tn*558*, 11 aggregated in two locations in Bavaria (74, 75, 127, 45, 46, 141, 144, 145, 152, 158, 174; Fig. 3). In addition, several isolates that were collected in nearby locations showed identical resistance profiles. Out of five isolates carrying the biocide resistance gene *qacG* on a plasmid, two isolates each were found in Thuringia (151 and 164) and Brandenburg (166 and 178) (Fig. 3).

### Temporal correlations of typing results, virulence, and resistance properties were observed less often.

The total number of isolates per year varied from one (2014) to 45 (2008) (Table 4). From 2007 to 2019, most of the isolates investigated were obtained by 2010 (*n* = 116). In years with higher isolate numbers, a distribution all over Germany or at least over certain regions was observed (Table 4). Some isolates were even identified in the same zip code region in the same year. They also belonged to the same respective clusters, except for some isolates in North-Rhine Westphalia (23, 24, 29) and Thuringia (160, 161) (Table 4, Fig. 3).

Certain *spa* (t1197, t1451) and *dru* (dt10al, dt11ag, dt11ax, dt11c) types occurred only during the early years of our study period until 2010.

One isolate from 2007 and 22 isolates from 2008 were negative for the collagen adhesion-encoding gene *cna*. Considering later years, this was recognized only in 2018 for two LA-MRSA isolates. Furthermore, six of the eight isolates harboring either *seb* or *sek* and *seq* were from 2008 (*seb*, *n* = 1; *sek*/*seq*, *n* = 3) or 2010 (*seb*, *n* = 1; *sek*/*seq*, *n* = 1) and, thus, were identified only in the early years of our study period.

The presence of specific AMR determinants was overall not limited to certain years. Exceptions were the genes *vga*(A)_LC_ on a plasmid (2008 to 2009), *spc*_V_ as part of Tn*560* together with *lnu*(B) and *lsa*(E) (2018), and *spd* on a plasmid (2008). However, in each case only two LA-MRSA isolates carrying the respective gene(s) were found. Isolates harboring a *dfr* gene were detected during the whole study period. Until 2015, mainly *dfrK* was identified. However, since 2016 the dominant variant apparently changed so that more *dfrG*-positive than *dfrK*-positive LA-MRSA isolates were found. In 2018 and 2019, *dfrK* was not detected. The isolates carrying *qacG* on a plasmid from nearby locations in Thuringia and Brandenburg were collected in later years of the study period (2016 to 2019).

## DISCUSSION

Conventional systems still dominate among swine farms in Germany (https://www.giscloud.nrw.de/arcgis/apps/storymaps/stories/5e62a2b3316a45e18a356d7d6a6afeae). They have shown high percentages of LA-MRSA-positive samples, with CC398 dominating by far, while LA-MRSA was reported to be nearly absent in the more rarely occurring organic farms (20, 39). More precisely, LA-MRSA-CC398 has been reported to colonize the animals asymptomatically in almost half of German swine farms (20, 40) and up to 86% of humans occupationally exposed to them (17, 40). Fortunately, extended human-to-human transmission happened rarely, and nasal LA-MRSA carriage was found in only 0.08 to 0.2% of humans at hospital admission in Germany as a whole (17). Nonetheless, numbers are notably higher in areas of high livestock density (17, 41). In this study, LA-MRSA-CC398 was also recognized to be widespread all over Germany, largely reflecting the pork production density (https://agraratlas.statistikportal.de/#). However, isolate submission to the national resistance monitoring program GE*RM*-Vet occurs on a voluntary basis and includes only isolates from diseased animals; hence, the program does not necessarily reflect the true prevalence of monitored pathogens.

The cgMLST analysis revealed close relationships between the LA-MRSA-CC398 isolates, as their allelic profiles varied in only 0 to 9.9% of 1,569 alleles included. The assignment of isolates to a cluster was based on a previous study stating that S. aureus isolates displaying ≥30 allelic variations should be considered unrelated (42). Next to the main cluster (cluster 1) comprising most of the LA-MRSA, new side clusters and singletons emerged over time, which points toward an ongoing diversification within the porcine LA-MRSA-CC398 lineage resulting in novel variants. Our discovery of new STs and *dru* types underlines this assumption. All *spa* types detected were related and typically associated with LA-MRSA-CC398 (20, 43–45). The *dru* types identified were also related, and many have already been known to occur in LA-MRSA-CC398 (dt3c, dt5e, dt6j, dt10q, dt11a, dt11ab, dt11af, dt11ap, dt11c, dt11v) (46–49). However, *dru* type dt9v was previously described only in MRSA-CC5 (48, 49) and the types dt10ao, dt11ax, and dt11ca only in methicillin-resistant Staphylococcus pseudintermedius (50–52). In accordance with previous literature (53), exclusively SCC*mec* type IV and V elements were found in the LA-MRSA-CC398 besides two truncated cassettes. The multitude of *spa* and *dru* types identified emphasizes the molecular diversity within CC398 that has already been reported and is still increasing (9, 20).

LA-MRSA-CC398 typically lacked major virulence-associated factors often found in community- or hospital-associated MRSA, such as TSS toxin or PVL (17, 54). Nevertheless, they harbored genes encoding hemolysins, exotoxins, enterotoxins, and an exfoliative toxin. Exfoliative toxin-producing S. aureus strains can induce greasy pig disease along with the usual causative agent Staphylococcus hyicus (55). This is of clinical relevance for LA-MRSA-positive swine farms, and one isolate of our collection originated from an animal with this medical history. The exfoliative toxin gene *eta* was also detected in an MRSA-ST398 from a hospitalized Chinese child (56). Interestingly, *eta* and enterotoxin genes were previously described as less frequent in LA-MRSA-CC398 (54, 57). All LA-MRSA carried the protease-associated genes *hysA*^VSaβ^, *paiB*, and *cfim*, whose prevalence in S. aureus was suggested to result in enhanced proteolytic activity (58). Consequently, these genes may be part of virulence-associated genomic traits that might account for the success of epidemic clones (58). However, so far, only the enhanced pathogenicity of LA-MRSA-CC398 due to *hysA*^VSaβ^ has been functionally confirmed (59).

LA-MRSA-CC398 typically exhibited a wide range of AMR to different classes of antimicrobial agents. In agreement with the literature (57), part of the isolates showed only resistance to β-lactams and tetracyclines (*n* = 20), while more comprehensive phenotypes were also recognized. The AMR profiles mirrored the amounts of antimicrobial agents dispensed in veterinary medicine in Germany (https://www.bvl.bund.de/SharedDocs/Pressemitteilungen/05_tierarzneimittel/2022/2022_PM_Abgabemengen_Antibiotika_Tiermedizin.html). Penicillins and tetracyclines account for the largest shares, followed by sulfonamides, polypeptides, and macrolides. These classes of antimicrobial agents are also commonly used on German swine farms (60, 61).

Next to AMR genes commonly found among staphylococci of animals (29, 57, 62), the novel *erm*(54) gene (22) and several other resistance genes were found in ≤10 isolates, including *erm*(T), *lnu*(A), *lnu*(B), *vga*(A)_LC_, *vga*(C), *lsa*(E), *aadE*, *spc*_V_, *spd*, *spw*, and *cfr*. Since *cfr* confers transferable resistance also to oxazolidinones, which are last-resort antimicrobial agents in human medicine, there is particular interest in the dissemination of this gene (63). As is known for LA-MRSA (62), several AMR genes mediating the same resistance phenotype were detected here, and many isolates harbored two or three AMR genes accounting for the same resistance property (Table 2). This might be due to an acquisition at different times and/or their location on MGEs carrying additional AMR genes (62). In fact, a large proportion of AMR genes was found to be part of small transposons or plasmids and, as such, might be transferred easily by horizontal gene transfer across strain, species, or genus boundaries. In addition to persistence of AMR genes in bacterial populations directly linked to the usage of certain antimicrobial agents, coselection processes due to more than one AMR gene or additional biocide and heavy metal resistance genes located on the same MGE need to be taken into account (62). MGEs harboring more than one AMR gene were also identified here. The characterization of SCC*mec* cassettes and possible, larger (multiresistance) plasmids among the 178 LA-MRSA isolates should be the subject of a follow-up study, because these elements could not be characterized completely here, as data from hybrid genome assembly were only available for a limited number of isolates.

Several characteristics of the LA-MRSA-CC398 were related to their phylogenetic affiliation to a cluster and/or their geographical origin. For example, many *spa* or *dru* types and AMR or biocide resistance genes were identified to a large extent or exclusively in isolates within the same cluster. The isolates within a cluster also often displayed similar or even identical AMR gene combinations. The main cluster 1, comprising most of the isolates, showed the greatest variety in molecular characteristics and AMR properties. Regional accumulations were most likely to be seen in clusters 2, 3, and 9. However, the latter two only comprised two isolates each. Isolates originating from the same zip code areas mainly belonged to the same cluster and/or were often identified in the same year. Certain *spa* and *dru* types were associated with specific areas in Germany. A spatial connection was also revealed for the detection of the biocide resistance gene *qacG* on a plasmid as well as the enterotoxin genes *seb*, *sek*, and *seq*. Regarding *sek* and *seq*, an association with cluster 2 was observed. Frequently detected AMR genes were spread all over Germany, but aggregation was also recognized in individual regions. More rarely occurring genes were observed in isolates from distant regions, although accumulation in individual areas was sometimes also revealed. In addition, similar or even identical AMR gene profiles were recognized in isolates originating from nearby regions. Temporal correlations of certain characteristics were recognized less often. Some *spa* or *dru* types were only seen in isolates identified during the early years. Isolates negative for *cna* or harboring one or two additional enterotoxin gene(s) besides *sep* tended to occur at the beginning of the study period. Next to a cluster-associated distribution, the AMR genes *dfrG* and *dfrK* most likely showed a time-dependent dissemination. The presence during limited periods was observed only for AMR or biocide resistance genes detected in small numbers. Finally, the exchange of genetic material between bacteria and their clonal dissemination enhanced by livestock trading activities, human occupational exposure, or emission of dust might be reasons for the isolates’ close phylogenetic relationships and the clonal and geographical correlations of characteristics outweighing temporal relations.

Studies of porcine LA-MRSA-CC398 have also been performed in other European countries (18, 64–69). The available information suggests that the AMR and virulence properties of those isolates are comparable to the characteristics of the LA-MRSA-CC398 isolates from Germany investigated in the present study.

Germany has established instruments providing antibiotic consumption data from human and veterinary medicine; however, region-specific data are needed (70) to evaluate the influence of possibly preferred agents on the distribution of individual resistance properties in specific areas and/or at certain times. Moreover, regarding all observed correlations here, it is worth noting that samples were not collected evenly over time and across the federal states in the GE*RM*-Vet program. Especially in the early years, the northern German federal states were overrepresented, and for Lower Saxony—the state with the highest swine density—too few isolates were included to get a coherent picture for all of Germany. Considering the lineage’s high diversification and transmission ratios and the intensity of livestock trade (9), a large-scale monitoring of LA-MRSA is needed so that intervention is possible in case novel, possibly more virulent and/or resistant variants are emerging. The prevalence of LA-MRSA will most likely not decrease (9), and the latest data available, covering part of our study period, even indicated an increase in the proportion of LA-MRSA in the German MRSA population, with up to 20% in “swine-dense” regions (71). If decolonization programs are targeted, a combination of measures concerning different husbandry factors needs to be considered (39).

In summary, this study uncovered diversity and population dynamics of the epidemic porcine LA-MRSA lineage in Germany over a 13-year-period. Clonal and geographical correlations of molecular characteristics, virulence, and resistance properties were identified. Full-scale LA-MRSA monitoring is needed to detect new emerging, possibly more dangerous clones and to prevent LA-MRSA transmission between livestock farms and introduction into human community or health care settings.

## MATERIALS AND METHODS

### Bacterial isolates.

In total, 178 MRSA-CC398 isolates from diseased swine were investigated in this study. The isolates originated from local diagnostic facilities in Germany and were included in the national resistance monitoring program GE*RM*-Vet during the years 2007 to 2019 on the basis of one isolate per swine herd. According to the background information from the diagnostic laboratories, 92 of the 178 isolates were presumably causative of the respective diseases (skin infections [*n* = 53], gastritis/enteritis [*n* = 9], musculoskeletal system infections [*n* = 7], septicemia [*n* = 6], central nervous system infections [*n* = 5], urinary-genital tract infections [*n* = 4], abscess/mammary lump [*n* = 2], staphylococcal infection [*n* = 2], abortion [*n* = 1], enterotoxemia [*n* = 1], greasy pig disease [*n* = 1], and polyserositis [*n* = 1]). One animal suffered from skin as well as central nervous system infection. Another 57 isolates were obtained from swine with respiratory tract infections and most likely represented colonizers of the respiratory mucosa, which were coisolated with other respiratory tract pathogens, such as Pasteurella multocida. For 25 isolates no background data were available except that the isolates originated from deceased animals. Finally, three isolates were probably only incidental findings and not causative agents of the respective diseases (streptococcal sepsis [*n* = 1], vitamin E and selenium deficiency [*n* = 1], and volvulus [*n* = 1]).

### Short-read and long-read sequencing.

All 178 LA-MRSA-CC398 isolates were subjected to short-read sequencing. Genomic DNA of 152 isolates was extracted using the QIAamp DNA Mini Kit (Qiagen, Hilden, Germany) with adaptions for staphylococci as described previously (72). The libraries were prepared using the Nextera XT DNA Library Preparation Kit (Illumina, Inc., San Diego, CA, USA) according to the manufacturer’s recommendations, followed by 2 × 300-bp paired-end sequencing in 40-fold multiplexes on the MiSeq platform with the MiSeq reagent kit v3 (Illumina, Inc.). Genomic DNA of the remaining 26 isolates was extracted using the HiPure Bacterial DNA Kit (Magen, Guangzhou, China). Here, the libraries were prepared with the KAPA Hyper Prep Kit (Kapa Biosystems, Boston, MA, USA). Sequencing was performed on the Illumina HiSeq X-Ten system (Annoroad Genomics Co., Beijing, China) and 2 × 150-bp paired-end reads with a minimum of 250-fold coverage were obtained for each isolate.

In addition, 12 LA-MRSA-CC398 isolates showing interesting characteristics were selected for long-read sequencing in order to generate complete, closed genomes. These isolates either held central positions within the different clusters of the cgMLST minimum spanning tree and/or harbored uncommon AMR genes/SCC*mec* cassettes and/or showed a—with the short-read sequences alone—not clearly identifiable SCC*mec* type. High-molecular-weight DNA was extracted with QIAGEN Genomic Tips 100G (Qiagen, Hilden, Germany) and sheared using g-TUBES (Covaris, Woburn, MA, USA) to an average of 10 to 15 kb. Libraries were prepared using the SMRTbell Express Template Prep Kit 2.0 (Pacific Biosciences of California, Inc., Menlo Park, CA, USA) according to the manufacturer’s protocol, “Preparing Multiplexed Microbial Libraries” (v07). Barcoded adapters were prepared with the Barcoded Overhang Adapter Kits 8A and 8B (Pacific Biosciences of California, Inc.). Equimolar pooling was calculated with the PacBio Express Microbial Multiplexing Calculator, followed by size selection with the BluePippin system (Biozym Scientific GmbH, Hessisch Oldendorf, Germany) using the high-pass option at 7 kb. Primer annealing and polymerase binding were done with sequencing primer V4 according to the SMRT Link Sample Setup (Pacific Biosciences of California, Inc.). Circular consensus sequencing reads were then generated by diffusion loading with 85 pM and an estimated insert size of 12 kb using one single-molecule real-time (SMRT) Cell (SMRT Cell 8M Tray, Pacific Biosciences of California, Inc.) and a Sequel II Sequencing Kit v2.0 (Pacific Biosciences of California, Inc.) on a Sequel II platform (2-h preextension, 30-h movie time; Pacific Biosciences of California, Inc.).

### Sequence assembly and annotation.

The 152 short-read sequences generated on the Illumina MiSeq platform were quality checked using FastQC v0.11.9 (https://www.bioinformatics.babraham.ac.uk/projects/fastqc/) and trimmed with Trim Galore v0.6.5 (RRID:SCR_011847). *De novo* assembly into contigs was carried out with Unicycler v0.4.9 (73). The 26 short-read sequences obtained from the Illumina HiSeq X-Ten system were assembled with SPAdes v3.12.0 (74) after quality control processing via FastQC v0.11.5 (https://www.bioinformatics.babraham.ac.uk/projects/fastqc/) and trimming with Trimmomatic v0.39 (75). In order to generate complete genomes of the 12 isolates subjected to long-read sequencing, the PacBio-HiFi long reads and Illumina short reads were hybrid assembled with Unicycler v0.4.9 (73) and the Flye algorithm in MaSuRCA v3.4.2 (76). In addition, a third assembly was performed with Canu v2.2 (77) using the PacBio-HiFi long reads. The generated genomes from MaSuRCA and Canu were polished using Pilon v1.2.3 (78) using the Illumina short reads. Subsequently, for all three generated genomes a consensus sequence was generated with Trycycler v0.5.3 (79). All assembled sequences were checked for errors with Geneious v11.1.4 (Biomatters, Ltd., Auckland, New Zealand) and annotated with Prokka v1.14.5 (80) as well as a subsystem technology server (RAST) (81) for further investigation.

### Molecular typing and phylogenetic analysis.

For all 178 LA-MRSA-CC398 whole-genome sequences, MLST, *spa* typing, and SCC*mec* typing were carried out using the MLST v2.0 (82), *spa*Typer v1.0 (83), and SCC*mec*Finder v1.2 (https://cge.food.dtu.dk/services/SCCmecFinder/) online analysis tools from the Center for Genomic Epidemiology (CGE). The *dru* types were identified according to the drutyping.org database (84) using the basic local alignment search tool (BLAST) function in Geneious v11.1.4 (Biomatters, Ltd., Auckland, New Zealand). Similar to *spa* typing, *dru* typing is a single-locus sequence-based typing method that, however, targets a region between the *mecA* gene and the adjacent IS*431* element in SCC*mec* cassettes of methicillin-resistant staphylococci. This region consists of mostly 40-bp direct repeat units (*dru*) which differ slightly in their sequences. The number and order of the different *dru* repeats define the *dru* type. As of 3 April 2023, 103 *dru* repeats and 579 *dru* types, comprising 1 to 23 repeats, had been assigned.

The 178 LA-MRSA-CC398 genomes were also subjected to phylogenetic analysis in Ridom SeqSphere+ v7.5.5 (85) using the S. aureus cgMLST approach (86). A minimum spanning tree was built based on a distance matrix of the core genome allelic profiles in order to illustrate the clonal relationships between the isolates. Overall, 1,569 of 1,861 potential target genes were included in the analysis by removing 292 columns with missing values from the comparison table. Isolates were assigned to clusters with a maximum threshold of 29 allelic differences (42).

### Investigation of virulence and AMR properties and MGEs.

In the 178 LA-MRSA-CC398 genomes, common virulence genes were identified with ABRicate v1.0.1 (https://github.com/tseemann/abricate) using the VFDB database (87) and with CGE VirulenceFinder v2.0.3 (88). Known AMR genes were located with ABRicate v1.0.1 using the NCBI AMRFinder tool (89) and the CGE ResFinder (90) databases. In individual cases of fragmented genes, the presence of AMR determinants was confirmed by PCR. Chromosomal point mutations conferring AMR were detected by applying PointFinder (91) as part of the CGE ResFinder v4.1 tool (90). The CGE PlasmidFinder v2.0.1 tool (92, 93) and MobileElementFinder v1.0.3 (94) were used to determine MGEs. All results were checked for errors with Geneious v11.1.4 (Biomatters, Ltd., Auckland, New Zealand), BLAST (95), and the UniProt knowledgebase (96).

### Antimicrobial susceptibility testing (AST).

AST by broth microdilution was performed in the GE*RM*-Vet program according to the recommendations of the Clinical and Laboratory Standards Institute (CLSI) for 29 antimicrobial agents using commercially available microtiter plates (Sensititre, Thermo Fisher Scientific, Waltham, MA, USA) (97, 98). Due to plate layout changes over the years, MICs for all 178 LA-MRSA-CC398 isolates are available for 18 antimicrobial compounds, including beta-lactams (oxacillin, amoxicillin/clavulanic acid 2:1, ampicillin, penicillin, cefoperazone, cefotaxime, ceftiofur, and cefquinome), tetracycline, macrolides (erythromycin, tilmicosin, and tulathromycin), lincosamides (clindamycin and pirlimycin), an aminoglycoside (gentamicin), a fluoroquinolone (enrofloxacin), folate pathway inhibitors (trimethoprim/sulfamethoxazole 1:19), and a glycopeptide (vancomycin). Isolates were classified as susceptible, intermediate, or resistant if breakpoints were available in the CLSI documents VET01S or M100 (97, 98) or in accordance with a previous study (3) (Table 3). Since several antimicrobial compounds were not included in the commercial microtiter plate layouts, the functionality of certain detected AMR genes was verified testing the growth of the corresponding isolates on Mueller-Hinton agar plates containing the respective antimicrobial agents at a specific threshold concentration. Despite the lack of CLSI-approved breakpoints, the functionality of the associated AMR gene was considered confirmed, in accordance with the sequencing data, for isolates that showed growth on plates with high concentrations of the antimicrobial agent (tiamulin, 2 to 8 mg/L [48]; kanamycin, 16 to 32 mg/L (99); streptomycin, 16 mg/L; spectinomycin, 128 to 256 mg/L [3]; apramycin, 16 mg/L [21]; trimethoprim, 8 mg/L [98]; florfenicol, 8 to 16 mg/L [99]). If available, CLSI-approved breakpoints or MIC values from previous studies were considered for the determination of threshold concentrations. In certain cases, up to two dilution steps lower than the information from the literature had to be tested. Moreover, screening for inducible macrolide/lincosamide resistance by agar disc diffusion (D-zone test) was conducted according to CLSI standards using discs containing erythromycin (15 μg; BBL Sensi-Disc, BD, Franklin Lakes, NJ, USA) and clindamycin (2 μg; Oxoid, Thermo Fisher Scientific, Waltham, MA, USA).

### Biocide susceptibility testing (BST).

BST for benzalkonium chloride was carried out by broth microdilution using commercial microtiter plates (Sifin Diagnostics GmbH, Berlin, Germany), which contained benzalkonium chloride in 12 2-fold dilution steps (0.000008 to 0.016%). BST performance followed a published protocol (100) that was modified as described previously (101).

### Data availability.

The whole-genome shotgun project comprising the genome sequences of 174 of the LA-MRSA-CC398 isolates included in this study has been deposited in GenBank under accession number PRJNA814867. The genome sequences of the remaining four LA-MRSA-CC398 isolates can be found within a previous project under accession number PRJNA842861. Isolate IDs and individual accession numbers are given in Data set S1.
